# Two Cases of Atopic Dermatitis Patients With Scleral Perforation After Recurrent Scleritis Induced by Scleral-Sutured Posterior Chamber Intraocular Lens Implantation

**DOI:** 10.7759/cureus.40153

**Published:** 2023-06-08

**Authors:** Akira Minamoto, Yosuke Harada, Tomona Hiyama, Hiromi Ohara, Yoshiaki Kiuchi

**Affiliations:** 1 Department of Ophthalmology, Hiroshima University, Hiroshima, JPN

**Keywords:** atopic dermatitis, scleral sutured iol fixation, scleral perforation, suture exposure, recurrent scleritis, intraocular lens implantation

## Abstract

This report describes two cases of atopic dermatitis patients with scleral perforation after recurrent scleritis induced by suture exposure after scleral-sutured posterior chamber intraocular lens (PC-IOL) implantation. The first patient was a 41-year-old man (case 1), and the second was a 46-year-old man (case 2). Both had a history of atopic dermatitis and scleral-sutured intraocular lens (IOL) implantation. Scleritis recurred at the suture site after scleral-sutured IOL implantation in both patients. Although the scleritis was controlled by topical and/or systemic anti-inflammatory drugs, the sclera was perforated in both cases because of exposure of the suture knots (after seven years in case 1 and after 11 years in case 2). In case 1, the superotemporal IOL haptic was also exposed over the conjunctiva, and in case 2, the ciliary body was incarcerated in the scleral hole with deformation of the pupil superonasally. Considering that there were no signs of severe intraocular inflammation, surgical intervention was performed in both cases. In case 1, IOL repositioning was performed with oral prednisolone cover at a dosage of 15 mg/day, starting two weeks prior to the surgery. The steroid dosage was gradually tapered off until two months after the surgery. In case 2, the scleral patch underwent without IOL extraction, and no steroid or immunosuppression cover was administered. There was no recurrence of scleritis after surgery in either case, and visual acuity was preserved in both cases. The scleral perforation that occurred after scleral-sutured IOL implantation in these patients was thought to be the result of recurrent scleritis caused by suture exposure and chronic mechanical irritation by a suture knot. The scleritis subsided without removal of the IOL by moving the suture site of the IOL haptic and covering the suture with a scleral flap or patch graft.

## Introduction

Transscleral suture fixation of a posterior chamber intraocular lens (PC-IOL) is one of the common intraocular lens (IOL) implantation techniques used in cases with no capsular support. IOL transscleral fixation has some postoperative complications, including cystoid macula edema, dislocation of the IOL, vitreous hemorrhage, choroidal hemorrhage, retinal detachment, and suture exposure [1]. There have been several case reports of scleritis causing thinning of the sclera and subsequent exposure of PC-IOL haptics. In these cases, the IOL needed to be removed because of endophthalmitis and purulent scleritis [1,2]. However, there have been no reported cases of successfully managing similar situations without IOL removal. Here, we report two cases of scleral perforation after recurrent scleritis that were triggered by suture exposure following scleral-sutured PC-IOL implantation and were successfully managed without the removal of the IOL.

## Case presentation

Case 1

**Figure 1:**
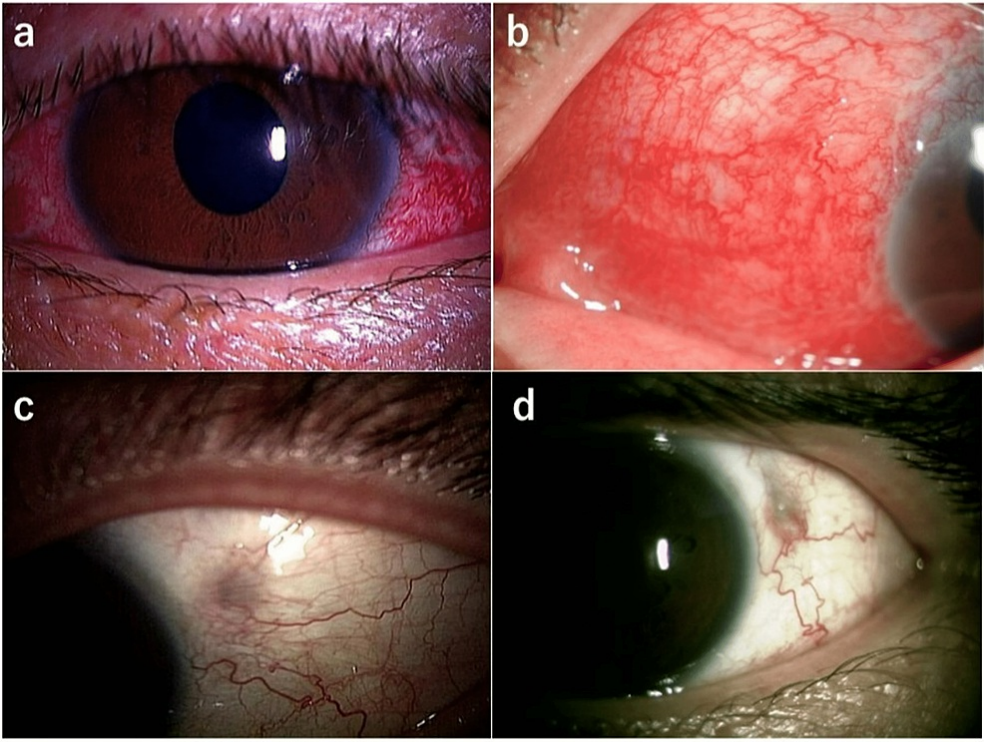
Findings on slit-lamp examination during follow-up after scleral-sutured posterior chamber IOL implantation in the left eye (case 1). (A) Three weeks after surgery, there was diffuse scleritis without exposure of suture or the IOL haptics. (B) One year after surgery, the patient developed a recurrence of severe scleritis and ocular inflammation that was treated with oral prednisolone and eye drops. (C) Fifteen months after surgery, there was a further recurrence of severe scleritis and ocular inflammation that was treated again with oral prednisolone and eye drops. (D) Four years after surgery. Although the suture knot could be seen under the conjunctiva, the IOL haptic was not exposed. IOL: intraocular lens.

The patient was a 41-year-old man who presented to a local eye clinic with blurred vision in the left eye after hitting his head. He was found to have ocular hypertension (intraocular pressure (IOP) of 39 mmHg) and a dislocated IOL in the left eye. He was treated with latanoprost/timolol maleate and dorzolamide hydrochloride eye drops and then referred to Hiroshima University Hospital. His best-corrected visual acuity (BCVA) was 20/12 in the right eye and 20/15 in the left eye. IOP was 18 mmHg on the right and 18 mmHg on the left. Slit-lamp examination showed inferior subluxation of the capsular bag-IOL complex in the left eye. Also, there were trace atopic blepharitis and injection of the left conjunctiva and grade 2+ cells in the left anterior chamber. He had a history of untreated atopic dermatitis (AD) that developed at the age of 24 years. He received treatment with local and systemic steroids for several years until the condition improved. Additionally, he developed an atopic cataract and underwent phacoemulsification combined with IOL implantation in the left eye five years earlier. Pars plana vitrectomy was performed with the extraction of the IOL and scleral-sutured IOL implantation. A single-piece polymethyl methacrylate IOL (NR-81K; Nidek Co., Ltd., Gamagori, Japan) designed specifically for suture fixation was used. The ab externo method [3] with 10-0 polypropylene suture was used to fixate the scleral suture. To avoid suture erosion, the suture knots were buried under the scleral pockets. Although the surgery was uneventful, he complained of redness and pain in the left eye three weeks postoperatively. Slit-lamp examination showed a diffuse injection of the left conjunctiva and grade 2+ cells in the left anterior chamber (Figure 1A). The suture knot that had been hidden under the scleral tunnel was not exposed over the sclera. He was diagnosed with scleritis in the left eye and treated initially with betamethasone eye drops. Although the scleritis and ocular inflammation improved after these treatments, they recurred two months postoperatively. On the other hand, since his referral to our clinic, there has been no exacerbation or deterioration of his AD. The diffuse scleritis subsequently settled after the reapplication of the same eye drops, and he was referred back to his local clinic. Seven months later, he was referred to our clinic again with a recurrence of scleritis and severe pain in the left eye. Diffuse scleritis was noted on the nasal side but there were no signs of suture or haptic exposure (Figure 1B). After confirming negative results for tuberculosis and syphilis, he was treated with the same eye drops and oral prednisolone 30 mg/day. The eye drops and oral prednisolone were gradually tapered off as the ocular inflammation improved (Figure 1C). He returned to the local eye clinic again four years later, at which time the IOL haptic was not exposed (Figure 1D).

**Figure 2:**
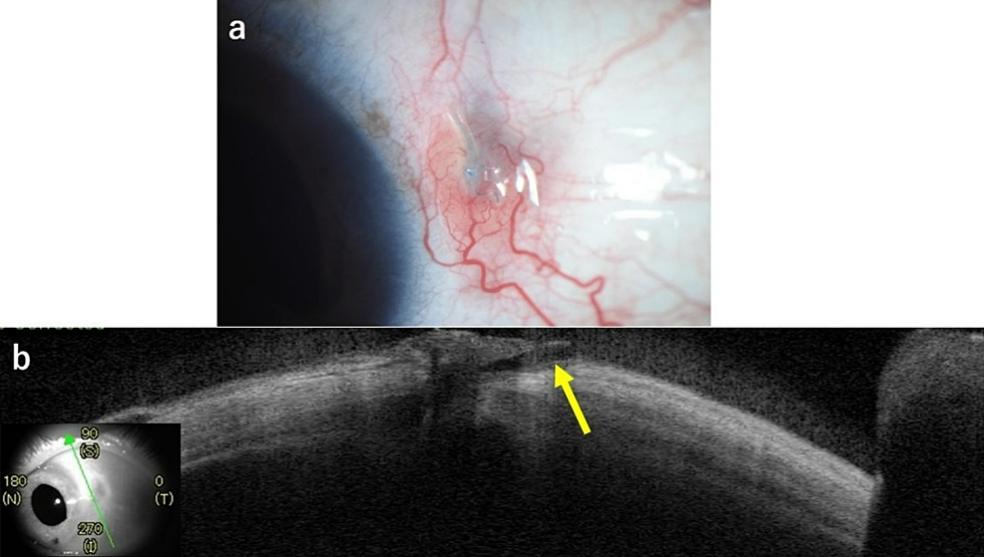
Findings in the left eye seven years after surgery (case 1). (a) Slit-lamp examination shows the temporal IOL haptic prolapsed over the conjunctiva and loosening of the suture. (b) Anterior segment optic coherence tomography (CASIA2, Tomey Corp., Nagoya, Japan) shows perforation of the IOL haptic with scleral thinning (yellow arrow). IOL: intraocular lens.

After a further three years (seven years after the last left eye surgery), he developed a foreign body sensation in the left eye and was re-referred to our facility. At presentation, his BCVA was 20/16 in both eyes with IOP of 20 mmHg in the right eye and 17 mmHg in the left eye. The temporal IOL haptic in the left eye had prolapsed over the conjunctiva and the suture was found to be loose (Figure 2).

**Figure 3:**
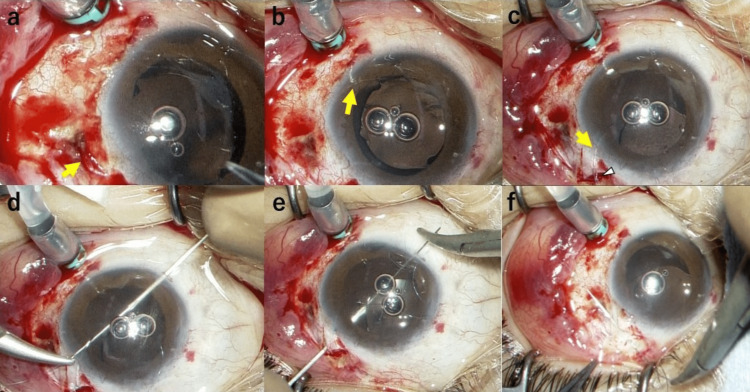
Intraoperative images (surgeon’s view) (case 1) (a) The temporal IOL haptic (yellow arrow) is exposed over the sclera. (b) The temporal IOL haptic (yellow arrow) was pulled back into the globe and positioned above the iris. (c) The temporal IOL haptic (yellow arrow) was pulled out through the side port, which was made at two o’clock on the corneal limbus. The haptic was secured using a long needle carrying a 10-0 polypropylene suture (arrowhead). (d) The long needle was engaged into the lumen of a 27-gauge needle and brought out through the opposite limbus (eight o’clock). (e) The 27-gauge needle was inserted through the scleral bed under a triangular half-thickness scleral flap that was made superotemporally from where the haptic was sutured, and the long needle was passed through to the opposite side. (f) The suture was tied to the scleral bed, after which the suture knot was completely covered by the flap. IOL: intraocular lens.

The IOL also tilted backward. The patient was diagnosed with scleral perforation caused by necrotizing scleritis. The initial plan was to extract the IOL and place a scleral patch to avoid further complications, such as endophthalmitis. However, the patient strongly preferred to retain his IOL. Therefore, the temporal IOL haptic was re-sutured to the superotemporal side where the sclera was intact (Figure 3). The surgery was performed with oral prednisolone coverage at a dosage of 15 mg/day, starting two weeks before the surgery and gradually tapered off until two months after the procedure.

**Figure 4:**
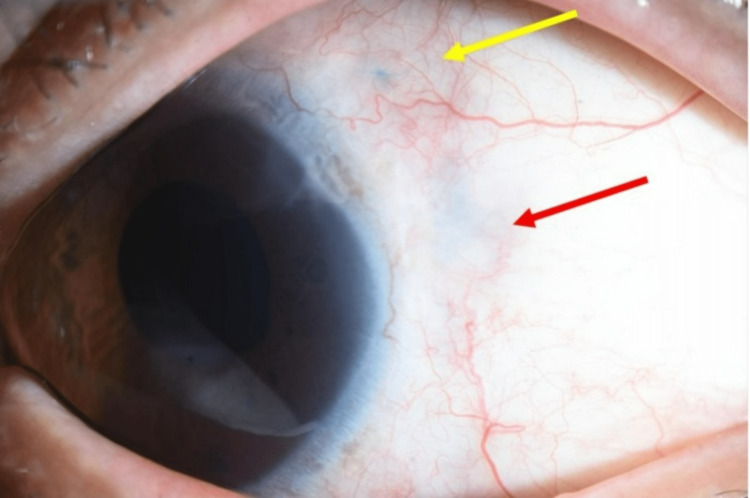
Photograph of the anterior segment of the left eye obtained at the last visit (3.5 years after the last surgery; case 1). There are no signs of recurrence of scleritis at the suture site. Red arrow: perforated site; yellow arrow: re-sutured site.

Although the IOL was shifted superiorly after the operation, the optic zone covered the pupil and his BCVA remained at 20/12.5 at three months after surgery. In the four years following the surgery, there have been no indications of scleritis recurrence or prolapse of IOL haptics, without the use of systemic steroids or immunosuppressive agents (Figure 4).

Case 2

**Figure 5:**
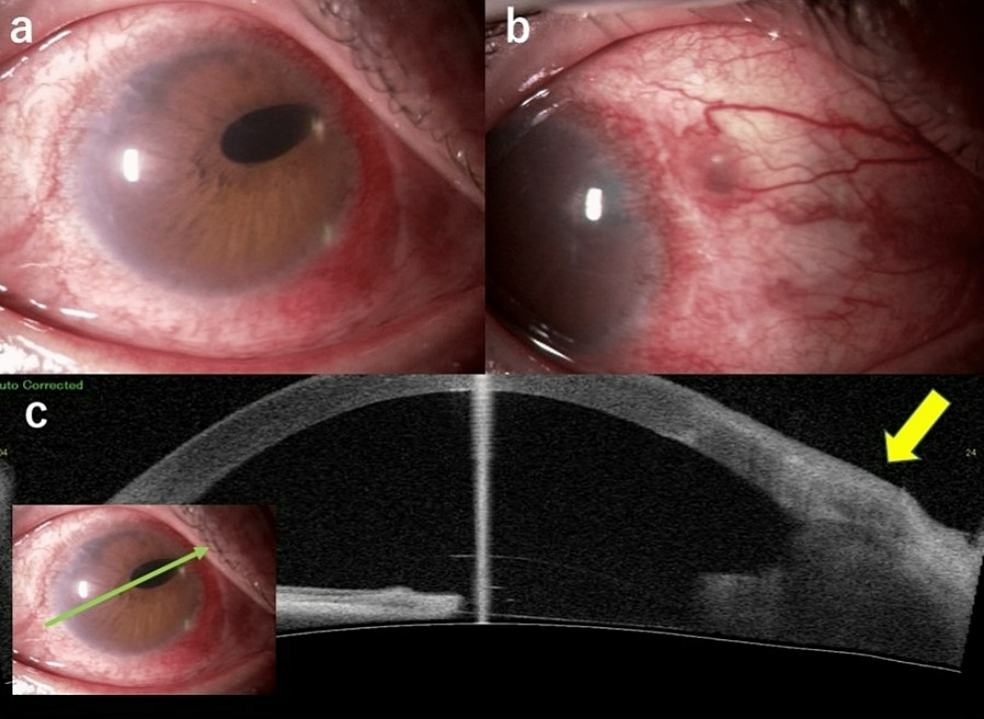
Photographs of the anterior segment of the right eye obtained 11 years after the surgery (case 2). (a, b) The pupil was deformed, and the ciliary body was incarcerated where the sclera was perforated. (c) Anterior segment optic coherence tomography (CASIA2, Tomey Corp., Nagoya, Japan) showed incarceration of the ciliary body in the scleral perforation site (yellow arrow).

The case was a 46-year-old man who was referred to Hiroshima University Hospital for right eye pain and redness. He had developed AD during his childhood, congenital nystagmus, and exotropia. His atopic blepharitis was not deteriorated since his first visit without local and systemic treatment. He also had a history of scleral-sutured IOL implantation [3] in the right eye for IOL subluxation seven years earlier. Decimal BCVA was 20/100 in the right eye and 20/100 in the left eye, and IOP was 14 mmHg on the right and 17 mmHg on the left. Slit-lamp examination of the right eye revealed trace blepharitis and scleral hyperemia where the nasal IOL haptic was sutured. The IOL was fixed and the pupil was round. Furthermore, there were no cells in the anterior chamber. The pain in the right eye resolved rapidly and the conjunctival injection improved after starting antibiotic eye drops, and he was referred back to his local clinic. Four years later, he complained of redness in the right eye, which did not improve on betamethasone eye drops. Therefore, he was re-referred to our clinic. The decimal BCVA was 20/125 in the right eye and 20/200 in the left eye, and IOP was 9 mmHg on the right and 11 mmHg on the left. There was mild hyperemia in the right eye that was relieved by phenylephrine eye drops. The superonasal suture was exposed over the injected conjunctiva where the sclera prolapsed, and the ciliary body was incarcerated (Figure 5).

**Figure 6:**
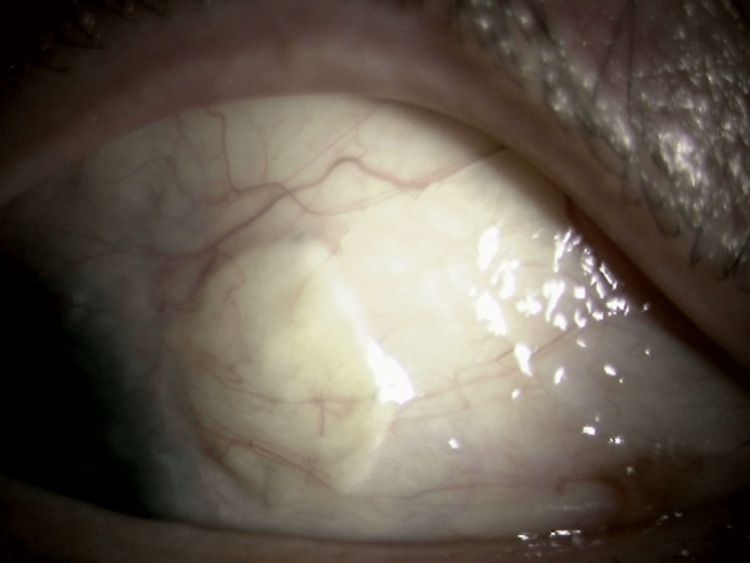
Photograph of the anterior segment of the left eye obtained at the last visit (four months after the scleral graft patch surgery) (case 2). The perforated site was covered with a preserved scleral patch graft and there were no signs of recurrence at the last visit.

The pupil was deformed to the superonasal side and grade 1+ cells were detected in the anterior chamber. The exposed suture was easily removed. A scleral patch was applied to the scleral perforation site a week later without IOL extraction given that there was no evidence of active scleritis. Intraoperative findings indicated that the ciliary body was incarcerated in the scleral hole where proliferative tissue was firmly adherent to the conjunctiva and sclera. There were no postoperative complications and his decimal BCVA was 20/125 in the right eye four months after the scleral graft patch surgery (Figure 6).

## Discussion

Transscleral fixation of a PC-IOL is one of the common methods used for lens implantation in patients with no capsular support. PC-IOL has several improvements over anterior chamber IOL and is associated with fewer postoperative complications [4]. Scleral perforation is rare following PC-IOL but is challenging to treat. This report describes two cases of scleral perforation following transscleral fixation of a PC-IOL and highlights the problem of scleral inflammation caused by suture exposure.

Transscleral fixation of a PC-IOL has some potential complications, including corneal edema, ocular hypertension, suprachoroidal hemorrhage, cystoid macular edema, retinal detachment, and suture-related problems [1]. Several suture-related complications have been reported, including lens tilt, lens dislocation, suture erosion, suture breakage, and endophthalmitis. Given that suture erosion increases the risk of endophthalmitis, immediate surgical intervention is recommended, either by covering the suture with a scleral or corneal graft or repositioning the suture knot under the sclera [1]. Uthoff et al. [5] reported that scleral erosion occurred in 19.7% of cases within one year of scleral-sutured PC-IOL surgery. Although burying the suture knot under a partial thickness scleral flap is known to be a useful technique for the prevention of erosion [6], Solomon et al. [7] found that the suture knot had eroded through the half-thickness scleral flap and conjunctiva in 17% of cases at six to 18 months after transscleral fixation of an IOL. In our cases, the scleral perforation occurred seven and 11 years after surgery, suggesting that the risk of this complication remains for a long time after surgery. Scleral perforation secondary to suture erosion is thought to be a rare complication of this technique but has the potential for devastating consequences. Ito et al. [2] reported two cases of scleral perforation by lens haptics. In both cases, the suture knot was not covered by the scleral flap and was seen below the conjunctiva. One case had severe iritis with scleral perforation that improved after extraction of the IOL; however, the other case developed endophthalmitis, leading to enucleation of the globe. Our two patients experienced several episodes of scleritis as a result of suture erosion that improved by systemic or local steroid therapy, indicating that these bouts of inflammation were associated with local mechanical stress that was mainly attributable to the exposed suture. These patients also had a history of AD. It is known that AD has synergistic activity with inflammation present in other organs, including other immune-mediated inflammatory diseases [8]. Therefore, mechanical stress with immune-mediated inflammation exacerbated by AD might cause scleral perforation. Scleral thinning around the perforation sites was limited in our patients and there were no signs of severe endophthalmitis. Therefore, we could leave the pre-implanted IOL in place and preserve vision in both patients.

Sutureless intrascleral PC-IOL fixation is simpler to perform than scleral-sutured PC-IOL implantation [9,10] and is a popular method nowadays. Both techniques have potential risks in terms of exposure to foreign bodies, namely, suture or IOL haptics [1,11-15]. While the frequency of scleral perforation and exposure of haptics is considered relatively low for sutureless intrascleral PC-IOL fixation, ranging from 0% to 3.0% in the early postoperative period [12,15,16], erosion of haptics is a notable complication according to a recent report by Pakravan et al. [17]. Theoretically, haptics placed in the scleral tunnel by intrascleral PC-IOL fixation can cause persistent mechanical stress to the sclera. Scleral perforation secondary to local scleritis via a mechanism similar to that described in these cases could be a common complication of intrascleral PC-IOL fixation. The conjunctiva and sclera where the IOL haptics are placed require careful monitoring after intrascleral PC-IOL fixation and scleral-sutured IOL fixation.

## Conclusions

Transscleral sutured PC-IOL fixation continues to be a common procedure in patients without capsular support, and suture-related complications cannot be completely prevented. Suture and/or IOL haptic exposure may lead to severe ocular inflammation and postsurgical infection. Therefore, close monitoring for suture and/or IOL haptic exposure is necessary to prevent further severe complications. If exposure occurs, immediate surgical intervention is required. The surgical approach is challenging, and the decision to perform it should be based on the severity of inflammation or the probability of subsequent endophthalmitis.
